# 

**Published:** 1906-03

**Authors:** 


Sixty-first Year.

Vol. LXI. MARCH, 1906. No. 8

### BUFFALO MEDICAL JOURNAL

Established 1845 by AUSTIN FLINT, M. D.

[illegible] OFFICE
[illegible] 1906

INDEXED

EDITOR:
WILLIAM WARREN POTTER, M. D.
ASSISTANT EDITORS:
WILLIAM C. KRAUSS, M. D. NELSON W. WILSON, M. D.
ASSOCIATE EDITORS:
JAMES WRIGHT PUTNAM, M. D. ERNEST WENDE, M. D.
JOHN PARMENTER, M. D. JOHN A. MILLER, Ph. D.
HARVEY R. GAYLORD, M. D. MAUD J. FRYE, M. D.
JOHN A. RAFTER, M. D.

